# Effects of Strength vs. Plyometric Training on Change of Direction Performance in Experienced Soccer Players

**DOI:** 10.3390/sports8110144

**Published:** 2020-10-30

**Authors:** Håvard Guldteig Rædergård, Hallvard Nygaard Falch, Roland van den Tillaar

**Affiliations:** Department of Sport Sciences and Physical Education, Nord University, 7600 Levanger, Norway; hovi_7@hotmail.com (H.G.R.); falch7@hotmail.com (H.N.F.)

**Keywords:** joint angles, kinematics, COD, contact time

## Abstract

The purpose of this study was to compare how 6 weeks of strength- vs. plyometric training, which were matched upon direction of motion and workload, influences change of direction (COD) performance. Twenty-one experienced male soccer players (age: 22.2 ± 2.7) were pair-matched into a strength- (*n* = 10) and a plyometric (*n* = 11) training group. CODs of 45°, 90°, 135° and 180° performed from either a 4 m or 20 m approach distance were compared before and after intervention. Results showed no significant difference between groups. Significant effects were only found within the plyometric training group (−3.2% to −4.6%) in 90°, 135° and 180° CODs from 4 m and a 180° COD from a 20 m approach distance. Individual changes in COD performances showed that with the 4 m approach at least 55% and 81% of the strength and plyometric training group, respectively, improved COD performance, while with the 20 m approach at least 66% of both groups improved performance. This study showed that the plyometric training program can improve most CODs, with angles over 90°, although this is dependent on the distance approaching the COD. Considering the limited time of implementing physical conditioning, in addition to regular soccer practice in most soccer environments, the current plyometric training program can be advantageous in improving CODs at maximal intensity.

## 1. Introduction

The development of competitiveness in soccer requires an emphasis on physical conditioning [1]. Among multiple determinant factors in soccer, the sport requires substantial effort to improve forceful and explosive movements, also known as “maximal actions” [2,3]. The change of direction ability is one of these maximal actions and are considered essential for success in most team and individual sports [4]. Sheppard and Young [5] defined it as a pre-planned rapid whole-body movement with changes in velocity and direction. Change of direction (COD) is referred to as agility when the movement is unanticipated and involves games-specific perceptual and decision-making components [6]. Furthermore it was shown that agility is a discriminating factor between higher and lower levels of play in sports, while the differences are less substantial regarding COD [7,8]. Despite this, there will occur situations in games where CODs are pre-defined [9]. As such, training COD speed can increase the chances of evading opponents, creating space and scoring goals [10,11]. 

Many studies have investigated the effects of different physical training forms on COD ability and COD as a phenomenon [4,12,13,14,15,16]. Most studies generalized COD as one discrete ability, while not accounting for the specificity that different COD tasks represent [4,13,17]. For example, Reilly, Williams, Nevill and Franks [1] found COD to be a discriminating factor between different levels of play in young soccer players but the COD test can be regarded as biased to linear sprinting [6]. Additionally, the biomechanical demands of COD have shown to be angle- and velocity dependent, which influences the technical requirements, kinetics, kinematics and muscle activation in the task [18]. Thus, practitioners should assess COD ability across a spectrum of different angles when assessing COD ability. 

In the literature, both strength- and plyometric training are popular methodologies that aim to develop physical aspects determining COD speed, which includes strength- and muscle power [4,19]. There is typically a restricted time to develop force in COD [19]. Thus, plyometric training which typically offers a high velocity training stimulus, due to a rapid eccentric to concentric muscle contraction [20,21] is specific to most COD movements [20]. Falch, Rædergård and van den Tillaar [15] reported in their meta-analysis assessing COD in court and field sports, improvements of 1% to 14% for plyometric training interventions and −2% to 12% for strength training interventions, where effect sizes varied from zero to very large. However, there is a need to expand the knowledge regarding strength- and plyometric training upon COD performance in soccer as most of the research was conducted in a young population [22] in which most participants were adolescents [23,24,25,26], which could be different from senior soccer players due to difference in training experience.

Consequently, the effects of these training methodologies are dependent upon the participants’ fitness characteristics, seasonal variations, training frequency, training volume and design and duration of the training protocol [15,26,27]. Among several factors stated, research supports the use of two training sessions a week for optimal adaptations to explosive sports specific skills for both strength and plyometric training [28,29] and it has been recommended that training exercises should be performed both unilaterally and bilaterally in multiple directions when training to improve COD ability [4]. Besides these important factors it is important to draw focus to which COD task being measured. 

Bourgeois, McGuigan, Gill and Gamble [13] clarified that different COD tasks were either force or velocity oriented, depending upon the approaching speed and angle of direction change. It has been suggested that modest COD angles of <90° are more velocity oriented, while greater angles of >90° are more force oriented. Dos’Santos, Thomas, Comfort and Jones [18] reported that the larger the angle of direction change is, the longer the contact times and the greater the ground reaction forces are and angles larger than >90° were shown to reduce nearly all momentum [30]. Therefore, longer times spent with slower muscle contraction velocities are expected in larger-angle CODs and faster muscle contraction velocities are expected in smaller angle CODs (<90°) [31,32]. Thus, maximal strength training may be more beneficial at improving force-oriented CODs, whereas plyometric training that requires faster muscle contraction velocities might be more beneficial in improving velocity-oriented CODs.

To the best of the authors’ knowledge, no studies have investigated a continuum of different angles and approaching speeds and how this is influenced by maximal strength training and plyometric training. Therefore, the main objective of the present study was to compare how strength- vs. plyometric training, which were matched in regard to direction of motion and workload, influences different COD performances during the off- and preseason. It was hypothesized that plyometric training would be more effective at improving velocity-oriented CODs, while strength training would be equally effective as plyometric training at improving force-oriented CODs. A second hypothesis was that groups combined will elicit significant improvements in most CODs due to changes in physical strength and power capacities that influences the kinematics in the COD step. Practical findings may aid how strength and conditioning coaches and practitioners utilize training to improve different CODs, as CODs will vary across different sports and by player position. Potential findings may also lead to a better understanding of how physical training influences kinematics in the COD step. 

## 2. Materials and Methods

### 2.1. Method

To investigate the effect of plyometric and strength training on velocity and force-oriented COD performances, A randomized controlled trial with pre- to post measurements was used, in which one group used strength training, while the other group employed plyometrics for a period of six weeks. The training groups were matched in terms of workload as measured by impulse (Δmv = ∫Fdt) based upon the study of *Ettema, et al.* [33] and direction of motion to determine its effect on time to complete different changes of direction tests that were the dependent variable. The independent variables were performance changes in strength and plyometric tests from pre- to post test, in addition to changes in kinematics measured in the COD step. 

### 2.2. Participants

Twenty-one experienced male soccer players from the 2nd to the 6th highest level play in the Norwegian soccer league participated in this study. Ten players participated in a strength training group (age: 22.2 ± 3.0 years, body mass: 77.1 ± 7.2 kg, height: 181.4 ± 6.0 cm) and eleven players participated in a plyometric training group (22.6 ± 2.6 years, body mass: 82.5 ± 7.3 kg, height: 182.3 ± 5.7 cm). Participants had at least 10 years of player experience in organized soccer. A minimum of two soccer training sessions a week in the regular season was required for participation, in addition to being familiar with strength and plyometric training. The participants had no injury or illness prior to familiarization to be included. Each participant was informed of the testing procedures and possible risks and written consent was obtained prior to the study. The study was conducted with the approval of the Norwegian Center for Research Data and conformed to the latest revision of the Declaration of Helsinki.

### 2.3. Procedures

Prior to the intervention study, the participants underwent two sessions of familiarization, where they performed a series of COD tests, maximal strength tests and plyometric tests. The pretest took place two to three weeks after the first familiarization day during the off- and preseason period. No intensive training was performed in the 36 h preceding testing and the participants ate a light meal two hours before testing. At pretest, body height and mass were measured before the participants dressed in inertial measurement unit-based body motion capture suits (Xsens Technologies B.V. Enschede, Netherlands). This was used to measure kinematic variables in COD and to check that knee joint angles in different strength tests did not differ at posttest compared to pretest. Afterwards, the participants performed a standardized warm-up protocol consisting of 5 min of general warm-up at a self-selected jogging/running speed followed by three runs of 20 m, performed at 60%, 70% and 80% of estimated maximal sprinting velocity with 60 s of rest in between. Then, a specific warm-up with four sprints of 15 m was performed. Each sprint was followed by a cut to the left and right with 65° and 110° angles of direction change, respectively, with 80% of self-assumed maximal effort and 1 min of rest in between. The COD tests applied in this study were based on a similar approach to that in Schreurs, et al. [34]. 

**Figure 1:**
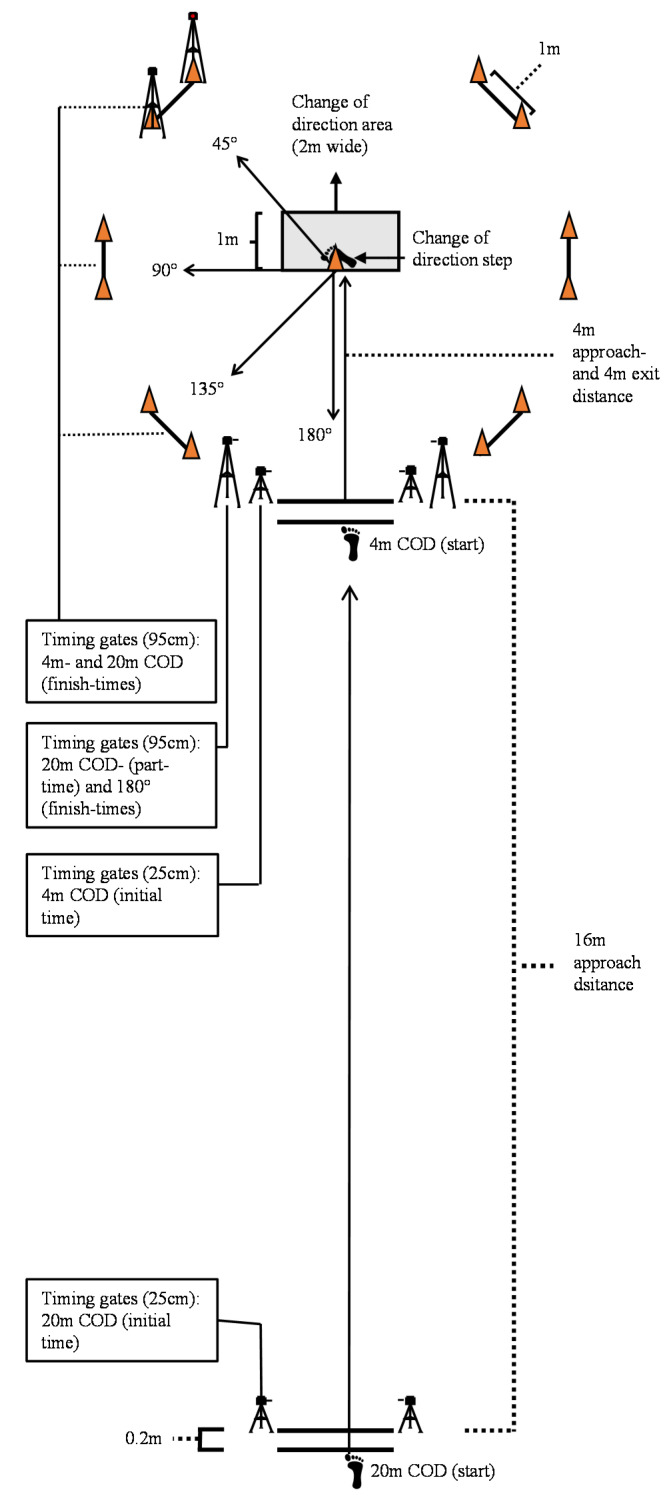
Change of direction (COD) test setup with approaches of 4 m or 20 m with timing gates on 4 m and 20 m with CODs of 45°, 90°, 135° and 180° followed by a 4 m sprint.

The participants had to approach the COD maneuver area from either 4 or 20 m with a left or right cut, where the angle of direction change was 45°, 90°, 135° or 180° (Figure 1). The athletes were instructed to complete each COD run as fast as possible. The COD test was performed on an indoor court surface (Taraflex Sport Evolution M 7.0 mm, Unisport, Finland). Each COD started with a standing start with the front foot placed 20 cm behind the timing gates (Brower Timing Systems, Salt Lake, UT, USA), which were 30 cm high and which were placed on each side of a 2 m-long line. Timing gates for measuring partial time and total time were 95 cm high. For an attempt to be approved, the participant had to perform the COD with both feet inside the COD area, without overstepping the rear end of the area or the turning cone (Figure 1), except for the 45° COD. The turning cone was removed when performing the 180° COD. The participants had one attempt at each condition but in the case of slipping or violations of the test requirements, one extra attempt was made. The participants had three to five minutes of rest between each run. Although it was rarely required, a test attempt resulting in a performance decrease of 0.1 s or more from the second familiarization day resulted in a reattempt to ensure maximum performance.

In addition to measuring COD times, joint kinematics were measured in 3D at 240 Hz (Xsens Technologies B.V. Enschede, The Netherlands). This system has been shown to be valid and reliable in these type of movements [35,36,37]. The calibration procedure was completed with participants performing the N-pose after inserting all possible anthropometric data listed in the Xsens software. Joint angles (hip flexion, hip abduction and knee flexion) was defined by joint angle displacement relative to the joint angles in static N-Pose. Together with the lean angle, which were define by the tibia angle relative to the floor, these two variables were analyzed from the right limb at the lowest center of mass displacement displayed in the COD step. 

Center of mass displacement, defined as the largest displacement of center of mass displayed in the COD step while the right foot was in contact with the ground, was also collected together with the contact time. Contact time was visually verified by deriving the first frame of initial ground contact to the last frame of ground contact in the Xsens software of the right leg during the COD step. This was completed to obtain more information about eventual changes in kinematics that could explain possible changes in COD performances from pre- to post-test.

After the COD tests, the participants had a 30 min break where they consumed a light serving of instant oatmeal (396 kcal). After the break, four maximal dynamic strength and five plyometric tests were assessed in randomized order for each participant. It was decided that both COD, strength and plyometric test were performed within one session due to the fact that soccer players are accustomed to perform multiple high-intensity COD actions, typically over 30−40 per limb during a game [38]. Unilateral exercises were performed with the participants’ dominant leg, defined by their preferred leg for kicking a soccer ball, which was the right leg for all participants. 

### 2.4. Strength Performances

Participants performed three different one-repetition maximum (1RM) back squat tests (Unilateral quarter squat, bilateral parallel squat and lateral squat), in addition to unilateral plantarflexion. The unilateral quarter squat was performed in a Powerline smith machine (PSM144X, Body-Solid, Forest Park, IL, USA) while the bilateral parallel squat and lateral squat was performed by lifting the Olympic barbell (20 kg) from a squat rack. At each 1RM attempt in the strength tests, a strength-experienced person was spotting the participants. 

The squats were approved for each participant at posttest, when a maximal variation of ±3° in the knee joint compared to pre-test was found, measured with the Xsens software. Before each 1RM squat test, the participants performed one set of two repetitions at approximately 50% of 1RM and one set of one repetition at 80% of 1RM with 1–2 min of rest after each set, like previous research [39]. When attempting 1RM, up to three attempts were made. The weight chosen to be performed was estimated based on near-maximal loads performed on the second day of familiarization. 

In consultation with each participant following successful 1RM attempts, the barbell load was increased by 2.5–5 kg until no further weight could be lifted. The researchers decided that 1RM was accomplished through one attempt if improvements were perceived to be unlikely trough additional attempts. This was based on the bar velocity visually controlled by the researcher, complied with participants self-perceived exertion. Participants had 3–4 min of rest between each 1RM attempt [40,41], which also applied to the rest periods when attending a new strength or plyometric tests. 

**Figure 2:**
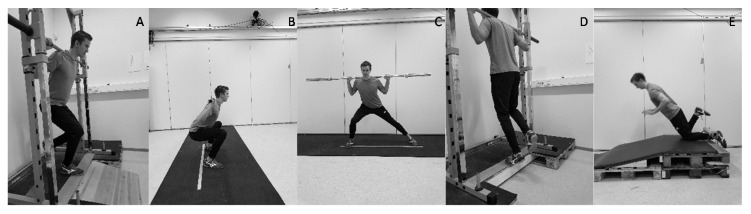
Performance of strength exercises. (**A**) Unilateral quarter squat. (**B**) Bilateral parallel squat. (**C**) Lateral squat. (**D**) Unilateral plantarflexion. (**E**) Unilateral Nordic hamstring (displayed for the purpose of the strength training program but not used as a measure of performance).

In the unilateral quarter squat test (Figure 2A), the toe had to be pointed forward on the edge of the platform and the participants had to reach a valid depth [42]. The nondominant foot was to remain passive, hanging slightly backwards. Participants were free to flex their hips but no rotation of the hip joint was allowed. In the bilateral parallel squat (Figure 2B), the participants were instructed to reach a parallel depth, which corresponds to a visualized line between the trochanter major and the patella, which was parallel to the ground. A barbell was placed on the upper trapezius; this was the barbell position that was used for all the squat exercises. There was no standardization regarding stance and the participants used a self-taught stance or a stance that was moderated by the researchers during the familiarization. 

In the lateral squat (Figure 2C), participants started with a hip-width stance followed by planting their dominant foot to the side. The lateral step had to be planted far enough so that the supporting limb could be fully extended. Unilateral plantar flexion required the participants to place the metatarsal bone over the edge of the gray platform (Figure 2D). They were “cued” to distribute the load on their big toe to prevent inversion of the ankle. The starting position was with the heel lowered on a wooden platform. On a signal, they extended the ankle maximally and were asked to hold that position for 2 s.

### 2.5. Plyometric Performances

Before each plyometric test (drop jump, unilateral CMJ, bilateral and unilateral hurdle jumps and skate jump), the participants performed two sets of the exercise at sub-maximal intensity with 1–2 min of rest in between. When testing, participants had three attempts with 2–3 min of rest between each test attempt, the best trial was used for analysis [43]. Jump height, ground contact time and reactive strength index (jump height in meters divided by contact time in seconds) were measured from the plyometric tests using a contact grid (Ergotest Innovation AS, Porsgrunn, Norway). 

**Figure 3:**
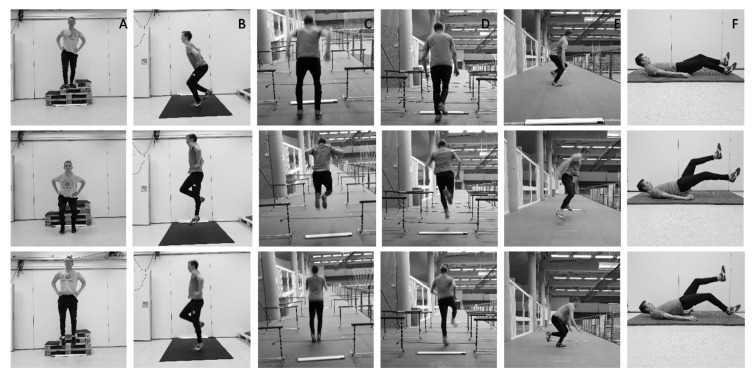
Different plyometric exercises. (**A**) Drop jump. (**B**) Countermovement jump. (**C**) Bilateral hurdle jump. (**D**) Unilateral hurdle jump. (**E**) Skate jump. (**F**) Lying kick (displayed for the purpose of the plyometric training program but not used as a measure of performance).

Drop jumps (Figure 3A) were performed with two legs with individualized drop heights of 30, 45 and 60 cm. The highest RSI score that the participants achieved on the second day of the familiarization was used to determine the optimal drop height [44]. Participants were instructed to keep arms akimbo, minimize the contribution of momentum created by forward leaning of the torso and mimic the instant of takeoff at landing [45,46], which was also practiced for unilateral countermovement jump (Figure 3B). Participants were further instructed to jump as high as possible with shortest contact time/SSC as possible in drop jump and unilateral countermovement jump respectively, in line with previous research [47,48]. 

Hurdle jumps were performed bilaterally and unilaterally (Figure 3C,D). The distance between each hurdle was 1.70 m for the bilateral condition and 1 m for the unilateral condition. Hurdle heights were standards of either at 20, 30, 40, 50 or 60 cm, which one of these heights applied to all hurdles within one test. This was based on the second familiarization day, which the height that athletes demonstrated the shortest contact time was used on the test day. The participants were instructed to jump over all hurdles as fast as possible, minimizing the contact time between [48,49]. The athletes performed three jumps within one series; the jump with the shortest contact time in three attempts was used for analysis.

In the skate jump, the participants started by placing their dominant foot on a marked tape. On a signal, they performed the exercise trying to reach maximal lateral distance, landing on their non-dominant foot with full control in the landing (Figure 3E). The performance was tracked by measuring the distance from the marked tape at the start to the middle of the heel used for landing. 

### 2.6. Training

After the test day, participants were evenly matched based upon their competitive level and performance in part-time and total time in the COD tests and assigned to either strength (*n* = 10) or plyometric (*n* = 11) training. However, one player from the strength training group got injured and was therefore excluded. During the intervention, participants were not allowed to train lower limb strength and plyometric exercises at their spare time, in addition to sprint, COD and deceleration training outside their regular soccer practice. For both the plyometric and strength training group, the training was performed in a controlled lab environment, supervised by a strength- and plyometric experienced researcher at each training session, providing participants individual feedback to ensure for both effort and technique in exercises. The participants were instructed to perform the strength exercises with a controlled eccentric phase of the lift [42]. In plyometric exercises, participants were encouraged to maximize performance with minimizing contact time and jump height. They were regularly provided feedback by contact mats, which functioned as a motivational tool. They were given cues such as fully extend their ankle when performing countermovement jump. The drop jumps and hurdle jumps were performed from the same height as deemed optimal at the day of testing. 

To match exercises from strength and plyometric training in terms of workload, impulse in the movements was calculated based on approaches from past research [33,50,51,52]. The total workload of each training program and their respective exercises was calculated and matched (Table 1). The workload for each training session equal to around 6000 Ns per training group per training session. The workload for the strength exercises was calculated by using a linear encoder (Ergotest Innovation AS, Porsgrunn, Norway) attached to a squat bar, measuring the maximum velocity in the movement. The workload for the plyometric exercises was estimated using indirect measures of flight time measured by a contact grid. The post-test occurred approximately one week after completion of the training program. 

The strength (Table 2) and plyometric (Table 3) training programs consisted of two sessions per week for six weeks with a progression from week to week. There was a minimum of 48 h of rest in between each training session. 

### 2.7. Statistical Analysis

To assess the effect of strength and plyometric training upon COD performances with different angles and approaches, a 2 (training group: independent groups) × 4 (degrees: 45‒180) × 2 (test occasion: pre, post) analysis of variance (ANOVA) with repeated measures was used on 4 m and 20 m approaches. In addition, percentage of change from pre- to posttest per group were calculated and per group repeated-measures ANOVAs were used to identify changes in 16 m part-times prior to the 20 m CODs (4 sprints) and performance changes in three strength exercises and five plyometric exercises. Besides the absolute strength performances, were the strength performances also normalized to body mass for each individual in order to present measures of relative strength. A two-way repeated-measures ANOVA (4 degrees × 2 times: pre, post) was also used to identify changes in kinematics for both groups combined from pre- to post-test. When significant differences occurred, Holm-Bonferroni post hoc tests were conducted to identify comparisons that were statistically significant. The level of significance was set at *p* < 0.05 and all data were expressed as mean ± SD after confirmation of the normal distribution using Kolmogorov Smirnov test. Independent sample t-test was conducted to check for differences in age, body, mass and height between groups. Effect size was evaluated with η^2^ (eta squared) where 0.01 < η^2^ < 0.06 constitutes a small effect, 0.06 < η^2^ < 0.14 constitutes a medium effect and η^2^ > 0.14 constitutes a large effect [53]. The analyses were carried out using SPSS Statistics v26 (IBM Corp., Armonk, NY, USA). Since no significant differences between different left and right COD test completion times at baseline (*p* ≥ 0.17) were found, further analysis was only conducted on left-side CODs. In these CODs the dominant leg (right leg for all participants) was used in the COD step and kinematics were analyzed on the dominant foot. 

## 3. Results

There were no statistical differences in age, body-mass and height between the groups at baseline (*p* ≥ 0.21). All strength and plyometric exercise performances improved from pre- to post-testing (*F* ≥ 3.214, *p* ≤ 0.05, η^2^ ≥ 0.167). However, a significant effect from pre- to post (group × time interaction) was found in seven out of eight strength and plyometric exercises (*F* ≥ 4.776; *p* ≤ 0.045, η^2^ ≥ 0.230), with the bilateral hurdle jump being the exception (*F* = 0.435; *p* = 0.519, η^2^ = 0.028). The strength training group improved more in all strength exercises (bilateral, unilateral and lateral squats) than the plyometric training group, while the plyometric training group improved more in all plyometric exercises (except for the bilateral hurdle jump; see Table 4).

A significant test occasion effect (*F* ≥ 7.3; *p* ≤ 0.015, η^2^ ≥ 0.27) was found for all COD times with 4 and 20 m approaches. Furthermore, a significant effect of COD degree (*F* ≥ 550; *p* < 0.001, η^2^ ≥ 0.98) upon the COD times was found. However, no significant time*degree interaction (*F ≤* 1.4; *p* ≥ 0.247, η^2^ ≤ 0.07) or any group interactions were found (*F ≤* 2.6; *p* ≥ 0.121, η^2^ ≤ 0.13). Post hoc comparison revealed that only the 4 m 45° and 180° and 20 m 180° COD times significantly decreased from pre- to post-test (Table 5) and that with increasing degree the COD times increased.

All COD times between the different degrees were significantly different on *p* < 0.05 level. Note that positive percentage of change means faster COD performance at the post test.

**Figure 4:**
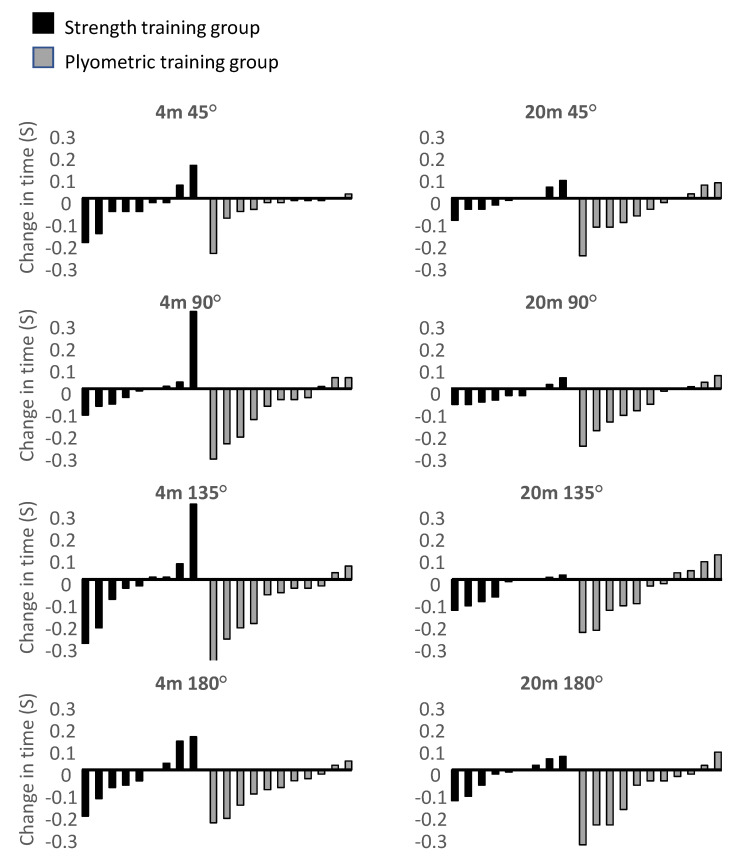
Individual changes from pre- to post-test (s) for each group with 4 m and 20 m approaches and four different COD angles.

However, when analyzed per group only significant decreases in COD times were found in 90°, 135° and 180° with the 4 m approach and 180° with the 20 m approach in the plyometric training group. Individual changes in COD performances within both groups showed that with the 4 m approach at least 55% and 81% of the strength and plyometric training group, respectively, improved COD performance, while with the 20 m approach at least 66% of both groups improved performance (Figure 4). 

**Figure 5:**
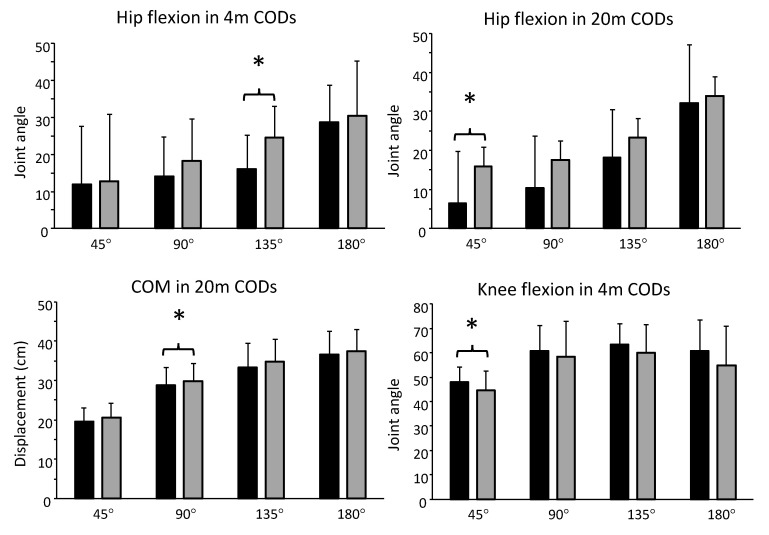
Mean (SD) at pre- and post-test of selected peak joint angles and center of mass height during COD with different angles. * indicates a significant change from pre- to post for the variable.

Significant changes in step and joint kinematics from pre- to post-test were found when both groups were combined (Table 6) for peak hip and knee flexion angle in 4 m CODs (*F* ≥ 10.7, *p* ≤ 0.032, η^2^ ≥ 0.43) and center of mass and hip flexion in 20 m CODs (*F* ≥ 5.8; *p* ≤ 0.035, η^2^ ≥ 0.29; see Figure 5). The remaining kinematic variables displayed no significant changes (*F* ≤ 3.12, *p* ≥ 0.103, η_p_^2^ ≤ 0.21).

## 4. Discussion

The aim of this study was to examine the effect of strength- vs. plyometric training that were matched in terms of workload and exercise direction of motion on CODs with different approaches, distances and angles. The main findings were that both training programs improved different CODs (Table 5), with no significant differences between groups. Changes in COD performances were accompanied by increased hip flexion, more displacement in the center of mass and less knee flexion (Figure 5). In addition, improvements in strength and plyometric performance after six weeks were related to the training program (Table 4). Strength performance was improved in the strength training group and plyometric performance was improved in the plyometric training group. 

Improvements in COD performance for both groups were partly comparable with previous studies and theory suggesting that strength training can induce better performance in strength-oriented CODs, while plyometric training can induce better performance in both strength- and velocity-oriented CODs [13,15]. Marked improvements were only found within the plyometric training group in the 90°, 135° and 180° CODs from 4 m and the 180° COD from 20 m, where improvement in percentage change varied from 3.2% to 4.6%. These increases were greater than in previous studies implementing plyometric training in short-duration CODs (<10 s) in a similar population [54,55]. The study points towards greater improvements in CODs induced by the plyometric training group, although the majority of the participants in both groups improved their performances in all COD tasks (Figure 4), which implies that both training approaches may be effective at improving different CODs. 

Changes in COD performances from a kinematic point of view can be explained by the fact that participants changed their way of altering joint mechanics from pre- to post-test. Sixteen out of 20 participants improved their performance in the 4 m 45° COD where less knee flexion was found at post-test. Therefore, it seems that participants stiffened the knee joint with minimal flexion. Generally, an increased knee angle during COD ensures greater braking and propulsive force application, typically demonstrated by stronger athletes [56]. However, a reduced knee flexion angle in 45° CODs will likely enable a more efficient force transmission between the knee and ankle joint, enabling greater exertion of vertical ground reaction force, deemed important for maintaining propulsion and velocity during directional changes of <45° [18].

Although approaching velocity was not quantified in this study, greater approaching velocity is expected in smaller-angle CODs and with increasing approach distance [18]. Havens and Sigward [57] reported no differences in ground reaction impulse between the penultimate step and the COD step in a 45° COD from a 7.5 m approach distance, indicating that braking is not an important factor in 45° COD as the penultimate foot contact plays an integral role in braking [58]. Therefore, the increased hip angle found in the 20 m 45° COD might be suboptimal as this will shift the center of pressure further and posteriorly away from the center of mass (COM) [34] and limits hip sagittal plane power [59]. This could explain why only 12 out of 20 improved their performance in the 20 m 45° COD as they failed to maintain velocity throughout the turn. 

Furthermore, increased hip angle and COM displacement were observed in 4 m 135° and 20 m 90° CODs. Turns of >90° have been shown to reduce nearly all momentum [30], meaning that exertion of horizontal/propulsive force will be prevalent in accelerating in a new direction. This can be accomplished by lowering COM [60] or increasing forward lean [61]. Fourteen out of 20 participants improved their performance in the 4 m 135° COD and those who improved had relatively large improvements, which were accompanied by relatively large changes in hip flexion angle from pre- (16°) to post-test (24.6°). Only 12 out of 20 participants improved in the 20 m 90° COD and the minor individual improvements may be explained by small changes in COM from pre- (28.8 cm) to post-test (29.8 cm). 

It is hard to draw any clear conclusion on how the two training programs influenced changes in kinematics. The strength training group improved their performance in three out of three strength exercises and in one out of five plyometric exercises. The plyometric training group improved their performance in five out of five plyometric exercises and in one out of three strength exercises. It is possible that participants had learned to apply greater ground reaction forces in the specific exercise range of motions trained, which is supported by the work of Rhea, et al. [62], who found increased levels of strength prior to a training intervention to be joint angle-specific. Alternatively, adaptations after training may also be explained by the fact that neuromuscular adaptations are velocity-specific [63,64] and specific to the contraction type and movement type trained [64]. This means that adaptations in muscle contraction velocity and force occur near or at the training velocity applied [65]. This was investigated by Loturco, et al. [66], who compared a group of young soccer players training with squat jumps with loads lower than their optimal power load and one group training with a load higher than their optimal power load. Results showed that the group training with a load lower than their optimal power load increased power production over the entire range (−20%, 0% and 20% of optimal power load). The group training with loads higher than the optimum load only increased at their optimal load condition and with loads 20% higher than optimal. Although there seems to be no clear consensus regarding adaptations in different parts of the force velocity curve following training, the strength training program is arguably suboptimal compared to the plyometric training group as the RFD in COD prevents players from expressing their maximal force capacity depending on the task [67]. 

These task-specific constraints may be exemplified by the fact that the strength training group displayed their greatest improvement in the 20 m 135° COD (−0.08 s). This was substantially better than the plyometric group (−0.01 s; see Table 4). This has a practical application considering that 135° directional changes represent a threshold, where a shift from a unilateral to a bilateral COD step typically occurs [18], thus players may approach the COD with a speed they feel they can tolerate based on their individual level of strength and perhaps modify the COD step technique based on their strength capacity. It is possible that players that underwent strength training approached the 20 m 135° COD with greater confidence in applying and tolerating the force with the 20 m 135° COD than the plyometric group. However, the effect in this task is unclear (*p* > 0.05) and needs further investigation. 

It should be mentioned that no differences were observed from pre- to post-test regarding contact time in different CODs. Considering the reduction in time taken to complete CODs, lower contact times in the COD step were expected as this has proved to be related to faster COD performance [68]. One possible explanation could be that the COD step is performed with a higher production of ground reaction force, which could result in higher concentric power output at push-off and thereby greater stride length, although this is dependent on the extent to which ground reaction force contributes to braking vs. propulsion.

The present study collected no kinetic data, which could have provided a more practical relevance and quantification regarding force production during COD and how this is related to the effect of the two training programs. Due to the variation in individual training responses and the limitation of only performing one attempt per COD it was difficult to compare results between the two training groups and the strength training was not appropriate for yielding statistical significance due to the lower sample size. In addition, further research with a test-retest correlation design is necessary to improve the findings of this study. Implementation of force plates sampling kinetic data from both the COD step and the penultimate step with several test trials is recommended to enable a better understanding of which phases of COD the training programs are affecting. Consequently, with the limitations raised, this should encourage future researchers to replicate parts of this study. 

## 5. Conclusions

The development of change of direction ability has become more specific. This research shows that there is some task-specific adaptation in COD depending on the angle of direction change and approaching velocity to the COD maneuver. In summary, both the strength and the plyometric training program, in particular, are useful for developing COD ability that requires angles of directional change of ≥90° and ≥135°, respectively, in mature male soccer players in six weeks. The plyometric training program can effectively be used by players that want to surpass or respond to opponents in anticipated situations where the angle of direction change is relatively sharp (>90°). Considering the limited time for implementing physical conditioning, in addition to regular soccer practice in most soccer environments, the current plyometric training program can be advantageous in improving CODs at maximal intensity. However, strength and conditioning coaches must carefully apply the training program based on the individual player, as previous work [15] has shown that a minimum level of maximal strength in the lower limbs is necessary for plyometric training to have an effect upon COD ability.

## Figures and Tables

**Table 1:** Matched exercises in strength- vs. plyometric group with corresponding muscles being targeted for physical overload and the sum of workload in these matched exercises per training group.

**Matched Exercises**		**Common Aspects**	
**Strength Training Group**	**Plyometric Training Group**	**Targeted Muscles from the Matched Exercises**	**Workload per Session**
Parallel squat, Unilateral squat & Calf Raise	Drop Jump, Unilateral CMJ & Hurdle jumps	Hip, knee and ankle extensor muscles	≈ 4250 Ns
Lateral squat	Skate jump	Hip abductor muscles	≈ 1650 Ns
Unilateral Nordic hamstring	Laying kick	Hamstring muscles	Peak EMG activity ≈ 75% of MVC (van den Tillaar et al., 2017)

Ns = Newton second, EMG = Electromyography, MVC = Max voluntary contraction.

**Table 2:** Periodized strength training program of six weeks.

**Week 1–3 (Session 1–6)**			
**Day 1**	**Intensity**	**Rest (s)**	**Series and Repetitions**
Unilateral quarter squat	85% of 1RM	180>	2 × 5 with each leg
Parallel squat	85% of 1RM	180>	3 × 5
Lateral squat	75% of 1RM	180>	3 × 6 with each leg
Nordic hamstring	Max braking	90>	2 × 5 with each leg
Unilateral plantarflexion	70% of 1RM	90>	3 × 8 with each leg
**Day 2**			
Unilateral quarter squat	80% of 1RM	180>	2 × 6 with each leg
Parallel squat	80% of 1RM	180>	3 × 8
Lateral squat	75% of 1RM	180>	3 × 8 with each leg
Nordic hamstring	Max braking	90>	2 × 5 with each leg
Unilateral plantarflexion	70% of 1RM	90>	3 × 8 with each leg
**Week 4–6 (Session 7–12)**			
**Day 1**	**Intensity**	**Rest (s)**	**Series and Repetitions**
Lateral squat	80% of 1RM	240>	4 × 4 with each leg
Unilateral quarter squat	80% of 1RM	240>	2 × 6 with each leg
Parallel squat	80% of 1RM	240>	3 × 6
Nordic hamstring	Max braking	90>	2 × 8 with each leg
Unilateral plantarflexion	75% of 1RM	90>	4 × 6 with each leg
**Day 2**			
Unilateral quarter squat	88% of 1RM	240>	2 × 4 with each leg
Parallel squat	85% of 1RM	240>	3 × 6
Lateral squat	70% of 1RM	240>	3 × 8 with each leg
Nordic hamstring	Max braking	90>	3 × 8 with each leg
Unilateral plantarflexion	75% of 1RM	90>	4 × 6 with each leg

1RM = 1 repetition maximum.

**Table 3:** Periodized plyometric training program of six weeks.

**Week 1–3 (Session 1–6)**			
**Day 1**	**Main Focus**	**Rest (s)**	**Series and Repetitions**
Unilateral CMJ	Height	90	5 × 1 with each leg
Drop jump	Reactive strength	60	10 × 1
Unilateral hurdle jump	Contact time	120	5 × 3 with each leg
Bilateral hurdle jump	Contact time	90	4 × 3
Skate jump	Reactive strength	90	3 × 6 with each leg
Laying kick	Reactive strength	90	2 × 5 with each leg
**Day 2**			
Drop Jump	Reactive strength	20	4 × 3
Unilateral CMJ	Height	60	6 × 1 with each leg
Bilateral hurdle jump	Contact time	60	6 × 3
Unilateral hurdle jump	Contact time	120	4 × 3 with each leg
Skate jump	Reactive strength	90	3 × 6 with each leg
Laying kick	Reactive strength	90	2 × 5 with each leg
**Week 4–6 (Session 7–12)**			
**Day 1**	**Goal**	**Rest (s)**	**Series and Repetitions**
Skate jump	Reactive strength	90	4 × 4 with each leg
Bilateral hurdle jump	Contact time	20	4 × 6
Unilateral hurdle jump	Contact time	120	4 × 3 with each leg
Drop jump	Reactive strength	60	8 × 1
Unilateral CMJ	Height	90	6 × 6 with each leg
Laying kick	Reactive strength	90	2 × 8 with each leg
**Day 2**			
Unilateral hurdle jump	Contact time	90	4 × 3 with each leg
Bilateral hurdle jump	Contact time	60	4 × 3 with each leg
Skate jump	Reactive strength	120	3 × 8 with each leg
Unilateral CMJ	Height	90	6 × 1 with each leg
Drop jump	Reactive strength	60	8 × 1
Laying kick	Reactive strength	90	3 × 8 with each leg

CMJ = Countermovement jump.

**Table 4:** Mean (±SD) of the different strength and plyometric exercise performances at pre and posttest for each group and between group comparisons.

	Strength Training Group			Plyometric Training Group			ANOVA
Exercise Variable	Pre	Post	Diff (%)	Pre	Post	Diff (%)	Effect: Group × Time (*p*)
Bilateral squat (kg)	113.6 ± 22.5	127.8 ± 19.5	12.5 *	130.0 ± 21.6	132.5 ± 13.0	1.9	0.021
Unilateral squat (kg)	88.3 ± 11.8	104.7 ± 11.3	18.6 *	98.7 ± 10.7	103.4 ± 12.0	4.7 *	0.023 *
Lateral squat (kg)	90.6 ± 15.4	106.3 ± 16.0	17.2 *	106.1 ± 15.4	104.3 ± 12.4	−1.7	0.001 *
Bilateral squat (kg/BM·kg)	1.47 ± 0.25	1.66 ± 0.22	12.7 *	1.57 ± 0.28	1.60 ± 0.18	2.1	0.028 *
Unilateral squat (kg/BM·kg)	1.15 ± 0.11	1.36 ± 0.08	18.2 *	1.19 ± 0.16	1.25 ± 0.15	4.8 *	0.019 *
Lateral squat (kg/BM·kg)	1.16 ± 0.16	1.36 ± 0.19	17.5 *	1.28 ± 0.21	1.26 ± 0.17	−1.6	0.001 *
Drop jump (RSI)	1.35 ± 0.27	1.31 ± 0.29	−3.2	1.27 ± 0.31	1.48 ± 0.29	16.8 *	0.015 *
Unilateral CMJ (m)	0.173 ± 0.039	0.185 ± 0.041	6.8	0.165 ± 0.029	0.196 ± 0.024	19.0 *	0.045 *
Bilateral hurdle jump (s)	0.173 ± 0.018	0.152 ± 0.022	−12.1 *	0.168 ± 0.023	0.152 ± 0.012	−9.5 *	0.435
Unilateral hurdle jump (s)	0.193 ± 0.092	0.188 ± 0.019	−2.7	0.197 ± 0.021	0.176 ± 0.017	−10.6 *	0.044 *
Skate-jump (m)	2.02 ± 0.16	2.04 ± 0.16	1.4	1.92 ± 0.19	2.02 ± 0.20	5.4 *	0.022 *

* indicates a significant effect on a *p* < 0.05 level.

**Table 5:** Change of direction time (4 m entry and exit time) at the different degrees with the 4 and 20 m approach at pre- and posttest for all and specified for each training groups.

Test	4 m 45°	4 m 90°	4 m 135°	4 m 180°	20 m 45°	20 m 90°	20 m 135°	20 m 180°
**All**								
Pretest	1.73 ± 0.15	2.06 ± 0.16	2.37 ± 0.17	2.48 ± 0.16	1.38 ± 0.11	1.84 ± 0.12	2.15 ± 0.14	2.30 ± 0.12
Posttest	1.69 ± 0.13 *	2.01 ± 0.15	2.30 ± 0.14	2.42 ± 0.13 *	1.36 ± 0.12	1.82 ± 0.09	2.11 ± 0.12	2.22 ± 0.11 *
Diff (%)	2.31%	2.43%	2.95%	2.42%	1.45%	1.09%	1.86%	3.48%
**Strength training group**								
Pretest	1.71 ± 0.17	2.03 ± 0.19	2.37 ± 0.20	2.44 ± 0.18	1.38 ± 0.10	1.84 ± 0.13	2.17 ± 0.08	2.32 ± 0.07
Posttest	1.67 ± 0.15	2.04 ± 0.19	2.35 ± 0.15	2.41 ± 0.15	1.34 ± 0.13	1.83 ± 0.11	2.09 ± 0.9	2.25 ± 0.10
Diff (%)	2.34%	−0.49%	0.84%	1.23%	2.90%	0.54%	3.69%	3.02%
**Plyometric training group**								
Pretest	1.74 ± 0.14	2.08 ± 0.13	2.37 ± 0.15	2.51 ± 0.15	1.38 ± 0.12	1.83 ± 0.11	2.13 ± 0.18	2.29 ± 0.15
Posttest	1.70 ± 0.12	1.99 ± 0.12 *	2.26 ± 0.13 *	2.43 ± 0.11 *	1.38 ± 0.11	1.82 ± 0.08	2.12 ± 0.14	2.20 ± 0.12 *
Diff (%)	2.30%	4.33%	4.64%	3.19%	0.00%	0.55%	0.47%	3.93%

* indicates a significant effect from pre- to post test for this COD time on a *p* < 0.05 level.

**Table 6:** Mean (±SD) step and joint kinematics at pre- and post-test for both groups combined.

		45° COD		90° COD		135° COD		180° COD		ANOVA
Variables	N	Pre	Post	Pre	Post	Pre	Post	Pre	Post	Effect: Time (*p*)
**4 m**										
COM disp. (cm)	17	17.7 ± 2.8	18.0 ± 4.3	25.4 ± 5.9	28.3 ± 5.3	31.2 ± 5.8	32.0 ± 6.9	34.5 ± 7.0	35.5 ± 9.5	0.200
Contact time (ms)	16	150 ± 23	160 ± 22	182 ± 37	204 ± 47	221 ± 42	238 ± 77	296 ± 82	304 ± 81	0.122
Hip flexion (°)	15	11.9 ± 15.8	12.8 ± 18.1	14.0 ± 10.8	18.3 ± 11.2	16.0 ± 9.2	24.6 ± 8.5	28.6 ± 10.2	30.4 ± 14.8	0.006 *
Hip abduction (°)	14	7.4 ± 5.2	9.0 ± 4.9	10.5 ± 6.0	10.1 ± 6.2	7.9 ± 7.5	10.3 ± 6.5	13.5 ± 7.9	10.3 ± 10.2	0.834
Knee flexion (°)	15	48.0 ± 6.0	44.5 ± 7.9	60.8 ± 10.4	58.2 ± 14.6	63.2 ± 8.7	60.1 ± 11.3	60.7 ± 12.7	54.7 ± 16.3	0.032 *
Lean angle (°)	11	28.1 ± 3.9	28.8 ± 4.9	38.0 ± 5.2	39.4 ± 2.3	43.1 ± 3.0	44.2 ± 4.3	47.1 ± 4.5	48.5 ± 3.7	0.279
**20 m**										
COM disp. (cm)	16	19.5 ± 3.5	20.5 ± 3.6	28.8 ± 4.6	29.8 ± 4.5	33.3 ± 6.1	34.6 ± 5.9	36.5 ± 5.9	37.3 ± 5.7	0.035 *
Contact time (ms)	15	150 ± 23	145 ± 21	186 ± 35	198 ± 52	216 ± 71	241 ± 76	335 ± 114	318 ± 90	0.735
Hip flexion (°)	15	6.5 ± 13.2	15.9 ± 16.8	10.4 ± 13.2	17.5 ± 13.7	18.2 ± 12.4	23.3 ± 10.7	32.1 ± 15.2	34.0 ± 16.7	0.030 *
Hip abduction (°)	14	9.8 ± 7.1	9.7 ± 4.9	10.0 ± 5.8	9.7 ± 6.0	11.7 ± 6.4	10.4 ± 8.0	16.7 ± 9.7	15.5 ± 7.0	0.562
Knee flexion (°)	15	49.0 ± 12.6	45.6 ± 12.2	59.6 ± 16.2	56.9 ± 15.7	61.4 ± 11.8	59.8 ± 14.8	60.2 ± 10.4	55.9 ± 17.0	0.185
Lean angle (°)	13	30.4 ± 5.6	32.7 ± 5.8	39.0 ± 4.7	40.1 ± 3.8	42.9 ± 3.7	44.4 ± 6.2	46.3 ± 6.6	48.2 ± 5.0	0.103

* indicates a significant effect from pre- to post test on a *p* < 0.05 level. COM disp. = center of mass displacement. Reduced N is apparent due to listwise exclusion of cases in ANOVA and the following post-hoc analysis, as there are some missing values in matching cases from pre- to post-test. The grey-shaded (highlighted) windows are further illustrated in Figure 5 to discriminate the specific significant pairwise comparisons. Note that sample size varies due to missing values.
